# Zheng Classification with Missing Feature Values Using Local-Validity Approach

**DOI:** 10.1155/2013/493626

**Published:** 2013-12-23

**Authors:** Yan Wang, Lizhuang Ma

**Affiliations:** ^1^School of Continuing Education, Shanghai Jiao Tong University, Shanghai 200240, China; ^2^Provincial Key Laboratory for Computer Information Processing Technology, Soochow University, Suzhou 215006, China; ^3^Department of Computer Science & Engineering, Shanghai Jiao Tong University, Shanghai 200240, China; ^4^Center of Traditional Chinese Medicine Information Science and Technology, Shanghai University of TCM, Shanghai 201203, China

## Abstract

Zheng classification is a very important step in the diagnosis of traditional Chinese medicine (TCM). In clinical practice of TCM, feature values are often missing and incomplete cases. The performance of Zheng classification is strictly related to rates of missing feature values. Based on the pattern of the missing feature values, a new approach named local-validity is proposed to classify zheng classification with missing feature values. Firstly, the maximum submatrix for the given dataset is constructed and local-validity method finds subsets of cases for which all of the feature values are available. To reduce the computational scale and improve the classification accuracy, the method clusters subsets with similar patterns to form local-validity subsets. Finally, the proposed method trains a classifier for each local-validity subset and combines the outputs of individual classifiers to diagnose zheng classification. The proposed method is applied to the real liver cirrhosis dataset and three public datasets. Experimental results show that classification performance of local-validity method is superior to the widely used methods under missing feature values.

## 1. Introduction

### 1.1. The Concept of Zheng Classification

Traditional Chinese medicine (TCM) is one of the most important complementary medicines used increasingly in the world [1]. Zheng classification enables the doctor to determine the stage that the disease developed and the location of the disease [2]. Zheng classification is the method of recognizing and diagnosing diseases by analyzing patient information based on TCM theories and the doctor's experiences [3].

**Figure 1:**
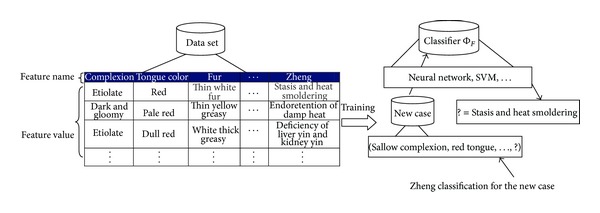
The process of intelligent Zheng classification.

In an attempt to achieve effective and objective standard of Zheng classification, various data mining approaches are used to construct the classifier on TCM dataset.  Figure 1 shows the process of intelligent Zheng classification.

### 1.2. Missing Feature Values: The Literature Review

In clinical practice of traditional Chinese medicine, feature values are often missing and incomplete cases. Missing feature values could be caused by various reasons, such as error of data measure, error of data understanding, erroneous human imputation, or restriction of data collecting [4, 5]. The performance of intelligent Zheng classification model in TCM is strictly related to the rate of missing feature values, but most common methods are short of the ability to solve the missing feature problem [4–6].

At present, the most common strategy for dealing with absent values is essentially to ignore them [7]. The cases with missing feature values are deleted before constructing the Zheng classification model [7]. Although improving the classification performance in some degree, deletion may discard some important information within the missing feature values, especially under the condition of insufficient TCM data. So deleting the data with missing feature values directly is difficult to meet the TCM clinical application.

Considering the shortcomings of the deletion method, imputation solution comes into being. Imputation is the substitution of a missing feature value with a meaningful estimate. Evidence theory is used to predict the missing feature values [8]. However, the evidence function should be learned in advance. The literature [9, 10] fills the missing feature by statistics method and Bayesian model, respectively. Nevertheless, these methods need to know probability distribution, which is difficult to be acquired in fact. In some applications, expert experience could be used to form the complete feature values. However, the prediction method for missing data by experts is subjective.

In recent decades, data mining imputation methods are beginning to attract much attention [11]. Logistic regression [12], subspace [4], neural network [13], and rough sets theory [14] have been applied to deal with missing feature values. These methods construct a predictive model to estimate the missing feature values from information within cases. However, imputation method will introduce new noise into cases, and the classification accuracy will decrease subsequently.

When dealing with the missing feature values, deletion and imputation methods will change the original dataset more or less. To avoid the problem of deletion and imputation methods, the literature [15–17] presents a selective Bayes classifier for classifying missing values with a simpler formula for computing gain ratio. Nevertheless, the method needs to satisfy the premise that features should be independent of each other. In TCM clinical practice, it is difficult to guarantee the characteristics of independence.

To overcome the limitation of the methods mentioned above, the proposed local-validity approach need not estimate the missing feature values or remove the deficient cases. It focuses on constructing intelligent Zheng classifier on the original cases directly. Firstly, the method finds the local-validity subset (LVS) within dataset and constructs the Zheng classifier on each LVS. Finally, the performance of each individual classifier is assessed and combined depending on the classification matrix to estimate the final output.

The rest of the paper is organized as follows.  Section 2 describes the dataset and the ideas of the proposed local-validity method. The experimental results based on the method are shown in Section 3. Finally, conclusion is given in Section 4.

## 2. Material and Methods

### 2.1. Description of Dataset

153 liver cirrhosis cases with three different Zheng classifications (i.e., stasis-heat smoldering zheng, damp-heat smoldering zheng, and liver-kidney yin deficiency zheng) have been collected from Shanghai University of Traditional Chinese Medicine. The dataset includes 52 cases with stasis-heat smoldering zheng, 61 cases with damp-heat smoldering zheng and 40 cases with liver-kidney yin deficiency zheng. Each case includes 40 TCM features selected by clinicians as the significant factors to identify the liver cirrhosis zheng.

Features are encoded using the four-value ordinal scales measured by the severity degree:

- 1 representing no corresponding symptoms;
- 2 for the normal level;
- 3 for the medium serious level;
- 4 representing the most serious.

Among all features, twenty-three features are missing in varying degrees. In this paper, the missing percentage *α* is defined as

(1) $$\begin{array}{c} \alpha =\frac{\left|U^{\prime }\right|}{\left|U\right|}, \end{array}$$

where |*U*′| denotes the number of cases with missing feature values and |*U*| denotes the total number of cases.

The list of these features and the corresponding missing percentage are shown in Table 1.

### 2.2. The Proposed Local-Validity Approach

**Figure 2:**
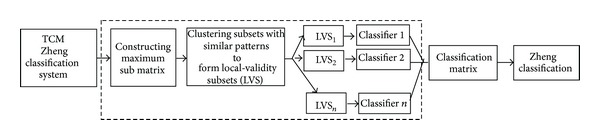
The overall view of the proposed local-validity approach.

As mentioned above, the local-validity idea overcomes the limitations discussed in the previous section. The flowchart of the proposed approach is shown in Figure 2. The subsequent subsections are organized as follows. First, the TCM zheng classification system with missing feature values is defined. Then, we describe how LVS is selected and how the individual classifier is trained on every LVS. Finally, we present how the individual classification results are combined to boost up the classification performance.

#### 2.2.1. Definition of Zheng Classification System

Zheng classification system with missing feature values in TCM can be viewed as a 3-tuple *S* = 〈*U*, *F*, *g*〉, where *U* is a nonempty finite set of cases and *F* is a nonempty finite set of features. For ∀*f* ∈ *F* and *x* ∈ *U*, *g*(*x*, *f*) denote the value that *x* holds on feature *f*. Then, in zheng classification system with missing feature values, ∃*x* ∈ *U* and ∃*f* ∈ *F* that satisfies *g*(*x*, *f*) = ∗. Here, we assume that the missing feature values are denoted by “∗.”

An example of zheng classification system with missing feature values is shown in Table 2.

#### 2.2.2. Finding Local-Validity Subsets

It is common that the number of missing feature values is *n* (*n* ≥ 1) in TCM clinical application. Based on the maximum sub-matrix theory, the missing feature values are considered as barrier points. The local-validity approach enumerates the maximum feature vector with complete values. Thus, the proposed method starts with a binary matrix *M* whose element is defined as

(2) $$\begin{array}{c} M_{i,j}=\{\begin{array}{cc} 1, & g\left(f_{i},x_{j}\right)\neq *,\\ 0, & g\left(f_{i},x_{j}\right)=*. \end{array} \end{array}$$

The element *M*_*i*,*j*_ = 0 if the *i*th feature is missing in the *j*th case.

The matrix *M* of the dataset presented in Table 2 is given as follows:

(3) $$\begin{array}{c} M=\left[\begin{array}{cccc} 0 & 0 & 1 & 1\\ 1 & 1 & 0 & 0\\ 1 & 1 & 1 & 1\\ 1 & 1 & 0 & 1 \end{array}\right]. \end{array}$$

Matrix *M* finds the maximum feature vector (MFV) that covers the most complete data. Each MFV identifies a local-validity pattern *P*; the formula of *P* is as follows:

(4) $$\begin{array}{c} \exists x\in U,\;\forall f_{i}\in \bar{P}\wedge \forall f_{j}\in \left(F-\bar{P}\right), \end{array}$$

satisfing (*g*(*f*_*i*_, *x*) = ∗)∧(*g*(*f*_*j*_, *x*) ≠ ∗) (*i* ≠ *j*).

Thus, the corresponding local-validity pattern *P* corresponding to Table 2 is

(5) $$\begin{array}{c} P_{1}=\left\{f1,f2\right\},\;\;P_{2}=\left\{f3,f4\right\},\\ P_{3}=\left\{f1,f2,f3,f4\right\},\\ P_{4}=\left\{f1,f2,f4\right\}. \end{array}$$

Each pattern *P* maps the corresponding LVS.

LVS is a collection of the cases that have no missing values for a specific feature subset and the collection of LVS includes all of the cases in the original data. The formula of LVS is as

(6) $$\begin{array}{c} \left(\forall x\in {\text{LVS}}_{k}\right)\wedge \left(\forall f_{i}\in {\text{LVS}}_{k}\right), \end{array}$$

satisfing *g*(*f*_*i*_, *x*) ≠ ∗, where LVS_*k*_ represents the *k*th local-validity subset.

**Figure 3:**
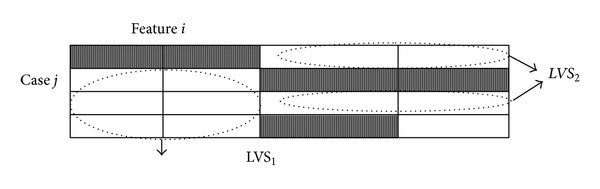
Example of local-validity subset.

Four LVS can be found from the dataset presented in Table 2 and Figure 3 shows two of them.

The process of finding local-validity subset can be described as follows.

1. For an original given dataset, generate matrix *M*.
2. In feature space, traverse matrix *M* to generate maximum feature vector.
3. For each feature vector, find the corresponding LVS.

As the feature missing percentage ascends, the number of LVS will increase. The large number of LVS will affect the computation complexity. Then, this problem will be translated into a clustering problem. LVS with the similar pattern will be merged.

#### 2.2.3. Clustering Local-Validity Subsets with Similar Patterns

It is desirable to cover the entire data with as few local-validity subsets as possible and obtain the overall best performance.

The preliminary research results [17] show that there are inherent consistencies between mutual information and subset aggregate.

Considering the cross entropy between two local-validity subsets,

(7) $$\begin{array}{c} \mu_{i,k}=-\log\left(\frac{1}{N_{i}N_{k}}\overset{N_{i}}{\underset{p=1}{\sum }}\;\overset{N_{k}}{\underset{q=1}{\sum }}G\left(x_{p}-y_{q},\sigma^{2}\right)\right), \end{array}$$

where *G* is the Gaussian kernel and *σ* ^2^ is the variance of Gauss function. *N*_*i*_ is the number of cases in subset LVS_*i*_ and *N*_*k*_ represents LVS_*k*_.

Then, the mutual information *I*(*i*, *k*) between LVS_*i*_ and LVS_*k*_ can be defined as follows:

(8) $$\begin{array}{c} I\left(i,k\right)=\mu_{i,k}\log\frac{N_{i,k}}{N_{i}+N_{k}}, \end{array}$$

where *N*_*i*,*k*_ represents the number of cases that belongs to two subsets at the same time.

The larger *I*(*i*, *k*) is, the stronger the correlation degree between LVS_*i*_ and LVS_*k*_ is. Based on *k*-nearest neighbor algorithm, the subset with strong correlation degree is clustered to form a new subset. In this paper, the *k*th cluster is represented by a set of LVS indices *Ω*_*k*_.

#### 2.2.4. Constructing the Zheng Classification Matrix

Once LVS is chosen, an individual zheng classifier is needed for each *Ω*_*k*_. In TCM zheng classification previous studies [18], the zheng classification matrix is proposed to merge the outputs of multizheng classifiers under the complete dataset.

Under missing feature values, in order to boost up the zheng classification performance, the complete degree *λ*_*i*_ is introduced into the zheng classification matrix *Y* to estimate the final output. Then, zheng classification matrix *Y* is updated as

(9) $$\begin{array}{c} Y=\arg\underset{w\in Y}{\max}\overset{k}{\underset{i=1}{\sum }}\lambda_{i}G_{i}^{w}, \end{array}$$

where *G*_*i*_^*w*^ represents the performance that a new case is diagnosed as *w* under the *Ω*_*i*_ local-validity subset.

## 3. Experimental Results

### 3.1. Local-Validity versus Other Methods on Liver Cirrhosis Dataset

To evaluate the performance of the proposed method, we carried out experiments on a real TCM liver cirrhosis dataset with missing data. Description of the dataset is presented in Table 1.

To analyze the improvement in zheng classification accuracy, three different methods are used to deal with the missing values.

The zheng classification accuracy is first estimated by simply removing the cases with missing values. Then, mean value imputation method is applied to impute missing feature values. Finally, the proposed local-validity approach is applied on the original dataset directly.

Considering the liver cirrhosis data is not sufficient, ten times 10-fold cross-validation is used for the assessment of classification performance. In cross validation, the data is split into ten approximately equal partitions and each in turn is used for testing and the remainder is used for training. That is, use nine-tenths for training and one-tenth for testing and repeat the procedure ten times so that, in the end, every case has been used exactly once for testing [19].

To get a reliable error estimate, the cross-validation process is repeated for 10 times, and the results are averaged [19].

The average classification accuracies are listed in Table 3. The best performance is emphasized using a boldfaced font.

As seen in Table 3, the performance of local-validity approach outperforms the deletion and imputation methods on liver cirrhosis dataset.

It should be pointed out that there are 23 feature values missing in original 40 features. Simply, deletion may introduce substantial biases, and imputation will introduce noise. With the increase of missing rate, problems of deletion and imputation will be more obvious. On the other hand, local-validity method constructs the zheng classification on the original dataset directly. The method can avoid the noise and biases problems.

### 3.2. Local-Validity versus Other Methods on Other Datasets

We also do experiments on three public datasets: lymphography, SPECT heart, and breast cancer.

Because these three datasets are complete, the diagnosis performance can be evaluated effectively. We replace randomly the feature value with “∗” based on different missing percentages *α* = {0,0.05,0.10,0.20} in these three public datasets. The results are shown in Table 4.

From Tables 4(a), 4(b), and 4(c), it can be seen that the performance of local-validity method is lower than that of deletion and imputation with *α* = 0.05. With *α* = 0.1 and *α* = 0.2, the proposed method performs well than other methods on three datasets. This shows that the performance of local-validity method is more stable than that of the other two methods, and the effect will be more obvious with the number increment of the missing cases.

In summary, the proposed local-validity algorithm is applicable to the dataset with small number of cases and a large percentage of missing values.

## 4. Conclusions

Although various machine-learning algorithms have been used to construct the zheng classification model in TCM, most of them deal with complete feature values. In fact, missing feature values are inevitable in TCM clinical application. Therefore, methods of constructing zheng classifier for missing data deserve more attention.

By analyzing missing data processing methods, this paper presents a local-validity approach for zheng classification with missing feature values. The proposed approach contains the following characteristics.

1. Instead of deleting or imputing the absent values, the proposed approach discovers the local-validity subsets from the original cases. Therefore, the proposed approach avoids the introduction of noise data.
2. Our method constructs zheng classifier on the original dataset directly and needs no assumption about the missing mechanism.
3. During the local-validity subset discovery phase, the formula for computation of the local-validity subset is presented. Then, the zheng classification matrix is described to combine the classification results of multi-individual classifiers.
4. Through experiments, we can conclude that the proposed method is an appropriate solution to missing feature values problems in TCM zheng classification. The results show that the proposed approach outperforms the deletion and imputation methods as the amount of missing feature values increases.
5. Further research is under way concerning the relationship between the scale of local-validity subset and classification accuracy in order to get the optimum diagnostic result.

## Figures and Tables

**Table 1:** Description of liver cirrhosis TCM dataset used in the experiment.

Feature name	*α* (%)
(1) Lassitude and fatigue	5
(2) Head heaviness	0.1
(3) Spontaneous sweat	10
(4) Nocturnal polyuria	0
(5) Depression	4
(6) Gingiva bleeding	0.2
(7) Blurred vision	3.1
(8) Reduced appetite	0
(9) Dry and bitter taste	0
(10) Abdominal pain	1
(11) Rib-side and flank distention and pain	2.3
(12) Low limbs puffy swelling	1.2
(13) Belching	0
(14) Yellow urine	0
(15) Scant urine	3.2
(16) Night sweat	1.1
(17) Sloppy stool	0.2
(18) Skin itching	2.1
(19) Skin bleeding	0
(20) Insomnia	0.3
(21) Limp aching lumbar and knees	0
(22) Tinnitus	0
(23) Hypochondriac distending pain	3.8
(24) Abdominal distension	0.1
(25) Yellow body	0
(26) Acid regurgitation	0
(27) Liver palm	0
(28) Dazzle	0.1
(29) Chill and cold limbs	2.1
(30) Constipation	0
(31) Vexing heat in the five heart	0.3
(32) Nose bleeding	0
(33) Rashness impatience and irascibility	0
(34) Fatigued and heavy limbs	0
(35) Dry eyes	4.1
(36) Epigastralgia	0.1
(37) Foul breath	0.2
(38) Yellow eyes	0
(39) Nausea vomit	0
(40) Spider naïve	0.1

**Table 2:** A dataset with missing feature values.

*U*	*f*_1_	*f*_2_	*f*_3_	*f*_4_
*x*_1_	∗	∗	1	0
*x*_2_	1	1	∗	∗
*x*_3_	0	1	1	0
*x*_4_	1	0	∗	1

**Table 3:** Performance comparison of three methods on liver cirrhosis dataset.

Classification accuracy (%)		
Deletion	Imputation	Local-validity
68.67	70.67	**80.33**

The bold values are used to emphasize the best Zheng classification performance.

**Table:** (a) Lymphography

*α*	Diagnosis accuracy (%)		
	Deletion	Imputation	Local-validity
0	**85.14**	**85.14**	83.7
0.05	**85.82**	83.78	84.02
0.10	81.21	81.08	**92.16**
0.20	78.92	77.43	**88.11**

**Table:** (b) SPECT heart

*α*	Diagnosis accuracy (%)		
	Deletion	Imputation	Local-validity
0	**82.4**	**82.40**	82.05
0.05	**82.28**	82.02	83.06
0.10	82	80.52	**85.25**
0.20	80.2	78.08	**86.06**

**Table:** (c) Lung cancer

*α*	Diagnosis accuracy (%)		
	Deletion	Imputation	Local-validity
0	**90.6**	**90.6**	90.05
0.05	89.25	**90.12**	89.17
0.10	86.23	83.37	**87.29**
0.20	81.67	82.02	**82.37**

The bold values are used to emphasize the best Zheng classification performance.
